# Supplementary material for: Ten-year trends in clinical characteristics and outcome of children hospitalized with severe wasting or nutritional edema in Malawi (2011–2021): Declining admissions but worsened clinical profiles
Source: PLoS One. 2024 Dec 26;19(12):e0311534. doi: 10.1371/journal.pone.0311534 (PMC11670969; doi:10.1371/journal.pone.0311534)
Supplement: S1 Fig — From a random start point (selected between 1 and 3), every 3rd file was selected for inclusion. One duplicated file was removed leaving 1,497 medical files included in the final analysis. (PDF) [file pone.0311534.s001.pdf]

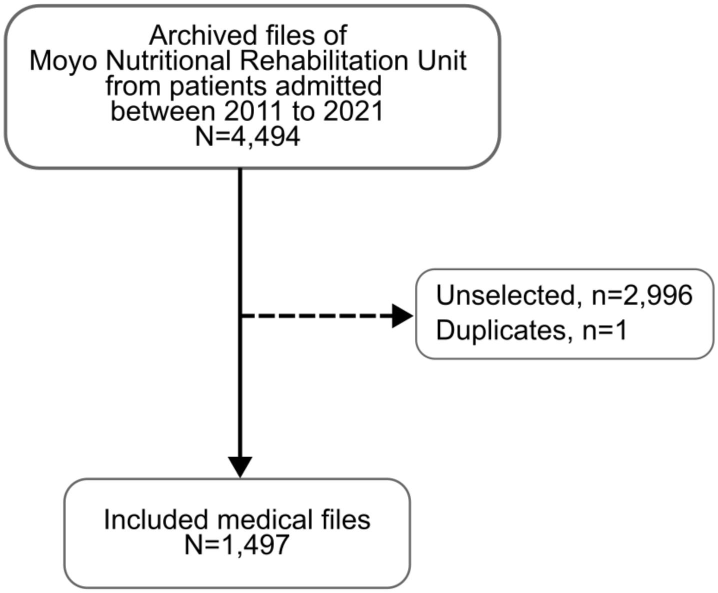

S1 Figure. Study flow chart representing selection procedure of medical files of severely malnourished children admitted at Moyo Rehabilitation Unit between 2011 and 2021. From a random start point (selected between 1 and 3), every 3rd file was selected for inclusion. One duplicated file was removed leaving 1,497 medical files included in the final analysis.
