# Supplementary material for: Ten-year trends in clinical characteristics and outcome of children hospitalized with severe wasting or nutritional edema in Malawi (2011–2021): Declining admissions but worsened clinical profiles
Source: PLoS One. 2024 Dec 26;19(12):e0311534. doi: 10.1371/journal.pone.0311534 (PMC11670969; doi:10.1371/journal.pone.0311534)
Supplement: S2 Fig — Frequency of edema (+), (++), (+++) as defined by the World Health Organisation presented across years by solid lines and black dots coloured as per legend. Linear and non-linear trends tested with general additive models. Grey dashed lines indicate linear fit with significance at right: n.s., non-significant, *p<0.05, **p<0.01, ***p<0.001. (PDF) [file pone.0311534.s002.pdf]

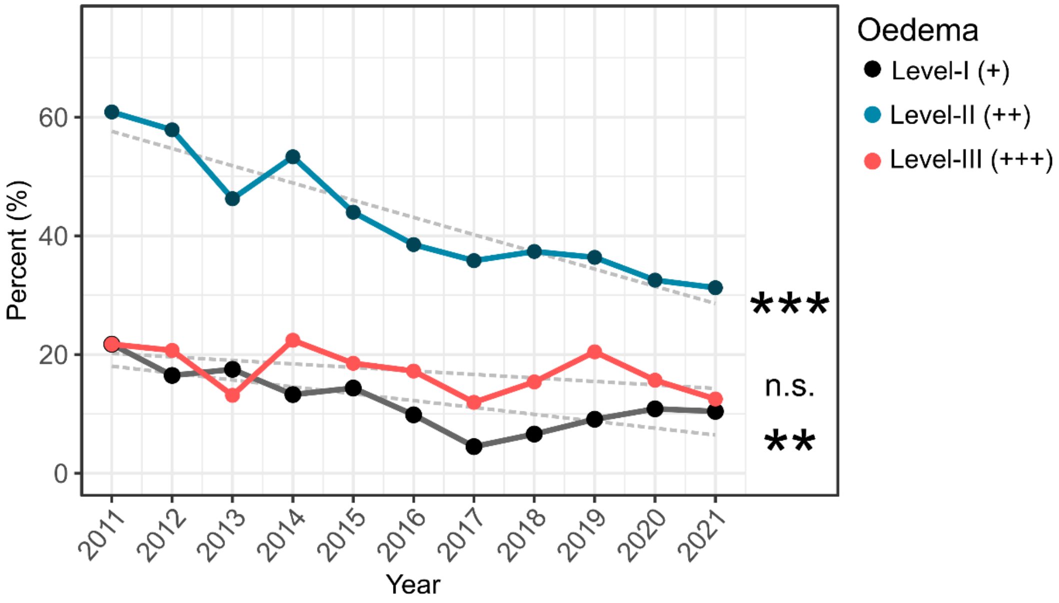

S2 Figure. Trends over 10 years in severity of oedema in children admitted at Moyo Rehabilitation Unit with nutritional oedema. Frequency of oedema (+), (++), (+++) as defined by the World Health Organisation presented across years by solid lines and black dots coloured as per legend. Linear and non-linear trends tested with general additive models. Grey dashed lines indicate linear fit with significance at right: n.s., non-significant, *p<0.05, **p<0.01, ***p<0.001.
