# Supplementary material for: Ten-year trends in clinical characteristics and outcome of children hospitalized with severe wasting or nutritional edema in Malawi (2011–2021): Declining admissions but worsened clinical profiles
Source: PLoS One. 2024 Dec 26;19(12):e0311534. doi: 10.1371/journal.pone.0311534 (PMC11670969; doi:10.1371/journal.pone.0311534)
Supplement: S3 Fig — A) HAZ across all children; B-D) MUAC, WHZ and WAZ in children with severe wasting; E-G) MUAC, WHZ and WAZ in children with nutritional edema. Solid midline (black) with dots shows median of growth metrics across years, top and bottom solid lines (red) are the high and low interquartile range. Linear and non-linear trends tested with general additive models. Grey dashed lines indicate linear fit with significance at right: n.s., non-significant, *p<0.05, **p<0.01, ***p<0.001. MUAC, mid upper arm circumference; WAZ, weight-for-age z-score, HAZ, height-for-age z-score (if < 24 months, length-for-age z-score); WHZ, weight-for-height z-score (if < 24 months, length-for-age z-score). (PDF) [file pone.0311534.s003.pdf]

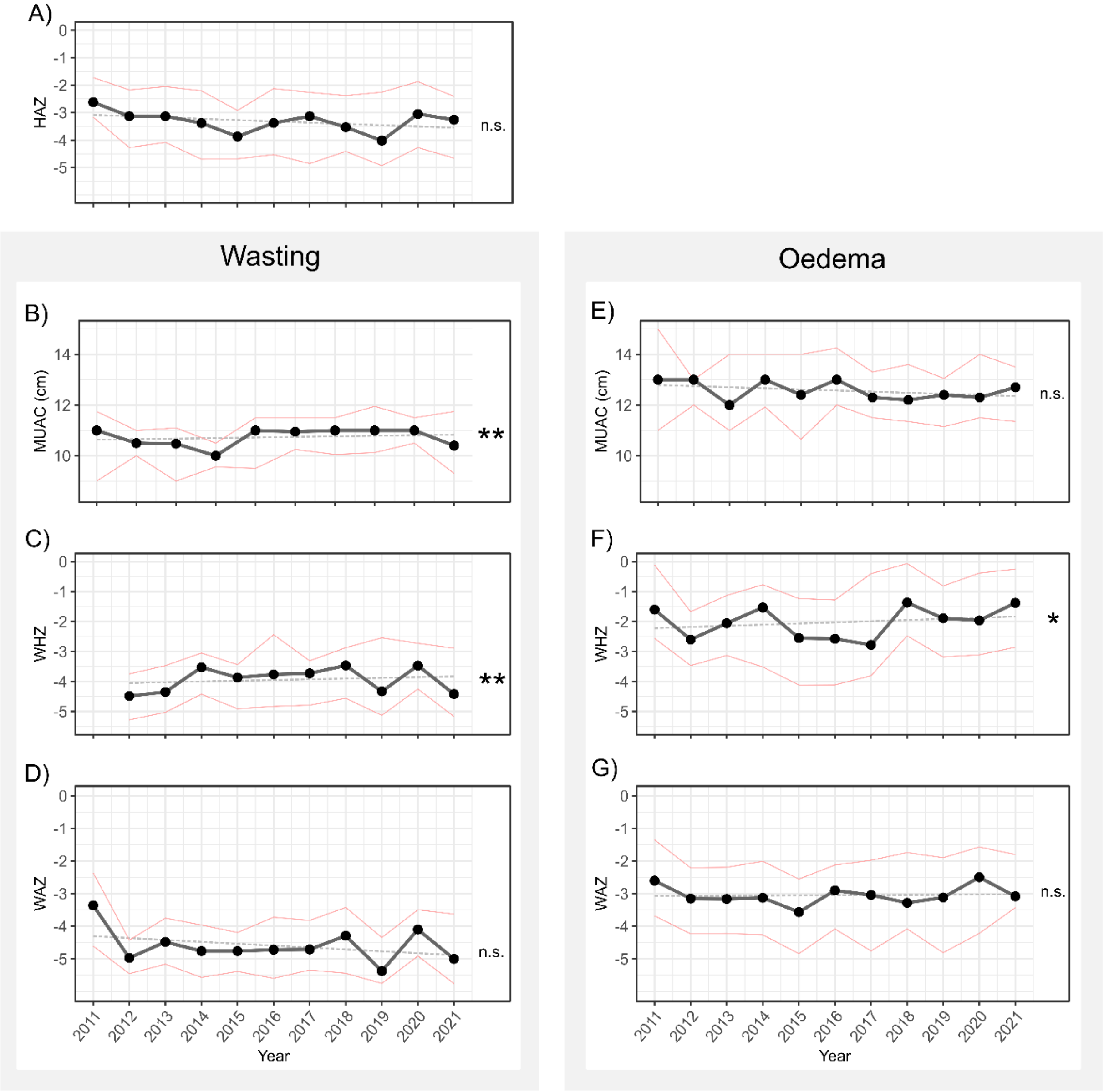

S3 Figure. Anthropometry and growth metrics of children admitted with severe wasting and/or nutritional oedema at MOYO NRU over the 10-year period. A) HAZ across all children; B-D) MUAC, WHZ and WAZ in children with severe wasting; E-G) MUAC, WHZ and WAZ in children with nutritional oedema. Solid midline (black) with dots shows median of growth metrics across years, top and bottom solid lines (red) are the high and low interquartile range. Linear and non-linear trends tested with general additive models. Grey dashed lines indicate linear fit with significance at right: n.s., non-significant, *p<0.05, **p<0.01, ***p<0.001. MUAC, mid upper arm circumference; WAZ, weight-for-age z-score, HAZ, height-for-age z-score (if < 24 months, length-for-age z-score); WHZ, weight-for-height z-score (if < 24 months, length-for-age z-score).
