# Supplementary material for: Ten-year trends in clinical characteristics and outcome of children hospitalized with severe wasting or nutritional edema in Malawi (2011–2021): Declining admissions but worsened clinical profiles
Source: PLoS One. 2024 Dec 26;19(12):e0311534. doi: 10.1371/journal.pone.0311534 (PMC11670969; doi:10.1371/journal.pone.0311534)
Supplement: S4 Fig — Frequency of A) Fever, B) Convulsion, C) Cerebral palsy, D) Rash, E) Jaundice. Linear and non-linear trends tested with general additive models. Grey dashed lines indicate linear fit with significance at right: n.s., non-significant, *p<0.05, **p<0.01, ***p<0.001. (PDF) [file pone.0311534.s004.pdf]

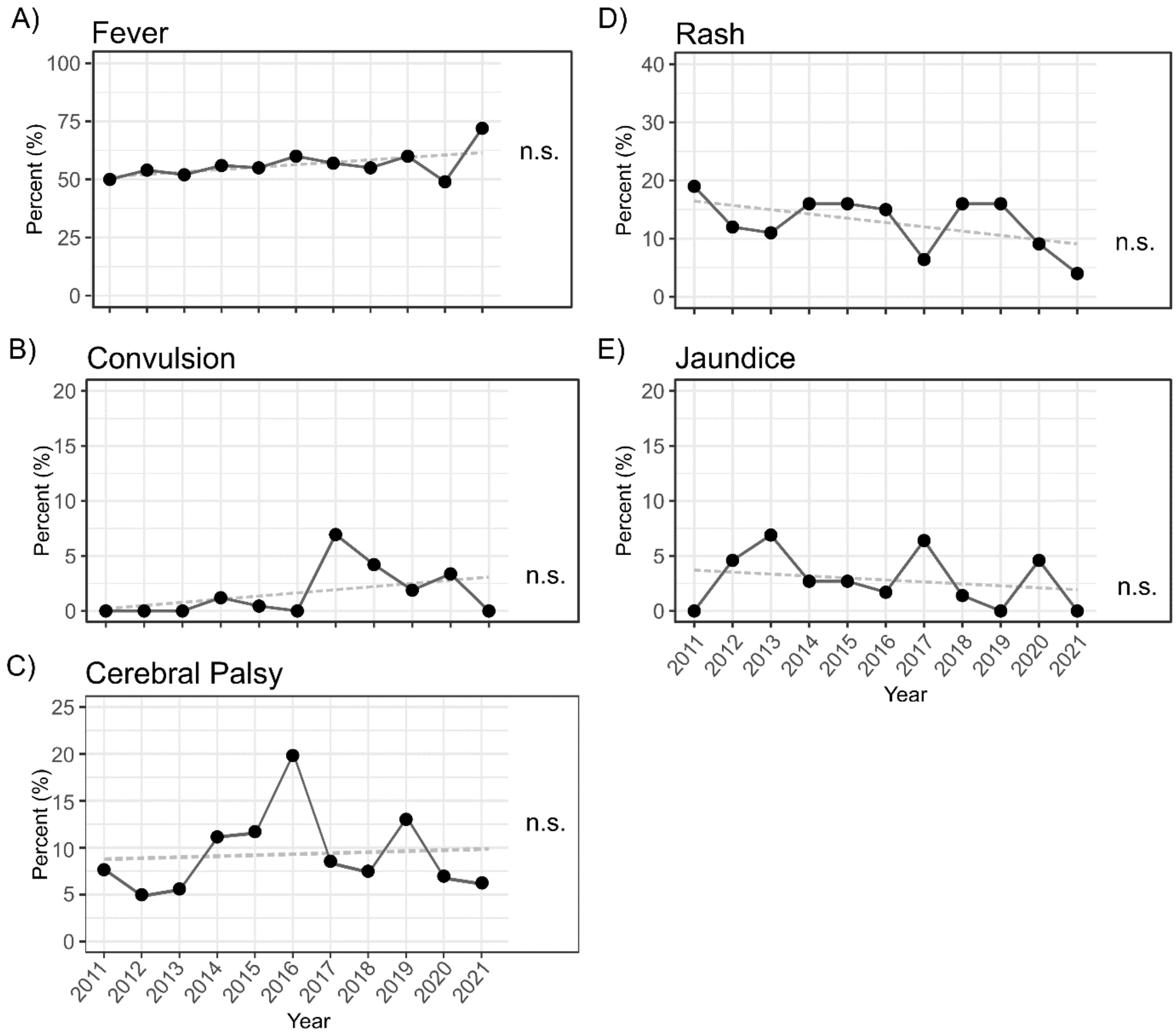

S4 Figure. Trends over 10 years in the Integrated Management of Childhood Illness (IMCI) danger signs presented by children with severe wasting and/or nutritional oedema admitted at Moyo NRU. Frequency of A) Fever, B) Convulsion, C) Cerebral palsy, D) Rash, E) Jaundice. Linear and non-linear trends tested with general additive models. Grey dashed lines indicate linear fit with significance at right: n.s., non-significant, *p<0.05, **p<0.01, ***p<0.001.
