# Supplementary material for: Ten-year trends in clinical characteristics and outcome of children hospitalized with severe wasting or nutritional edema in Malawi (2011–2021): Declining admissions but worsened clinical profiles
Source: PLoS One. 2024 Dec 26;19(12):e0311534. doi: 10.1371/journal.pone.0311534 (PMC11670969; doi:10.1371/journal.pone.0311534)
Supplement: S5 Fig — A) Dehydration, diarrhorea, and vomiting; and B) Dehydration, diarrhoea, vomiting very low WHZ (< -3.5 z-score). (PDF) [file pone.0311534.s005.pdf]

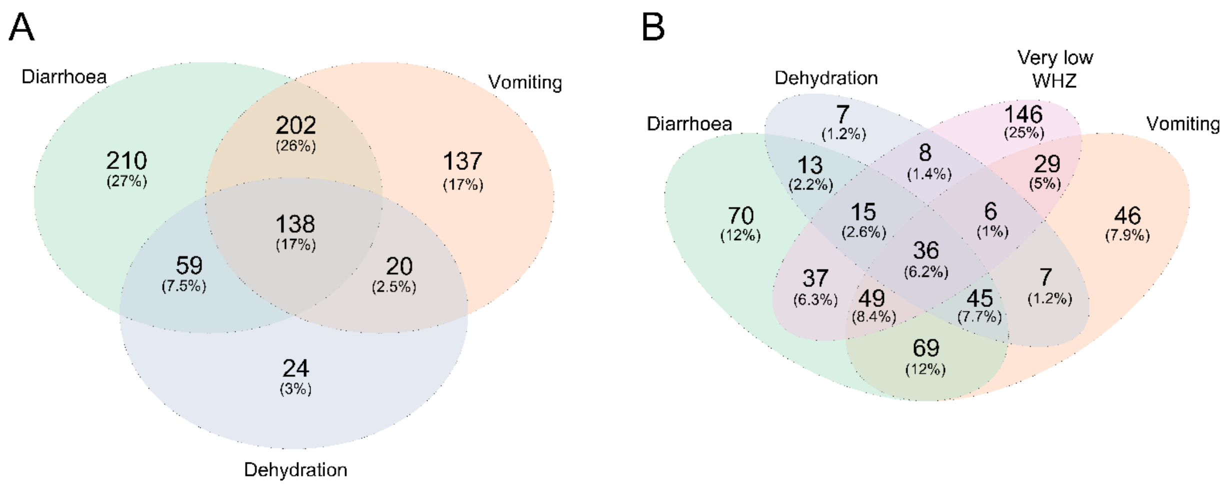

S5 Figure. Co-occurrence of symptoms related to gastroenteritis in children admitted at Moyo Rehabilitation Unit. A) Dehydration, diarrhorea, and vomiting; and B) Dehydration, diarrhoea, vomiting very low WHZ (< -3.5 z-score).
