# Supplementary material for: Ten-year trends in clinical characteristics and outcome of children hospitalized with severe wasting or nutritional edema in Malawi (2011–2021): Declining admissions but worsened clinical profiles
Source: PLoS One. 2024 Dec 26;19(12):e0311534. doi: 10.1371/journal.pone.0311534 (PMC11670969; doi:10.1371/journal.pone.0311534)
Supplement: S1 Table — Median and interquartile range or n (%) presented as appropriate. Prevalence of breastfeeding was calculated in children younger than 12 months of age. Linear and non-linear trends were tested with general additive models. (PDF) [file pone.0311534.s006.pdf]

S1 Table. Trends in age, sex, and breastfeeding over the 10-year period in children with severe wasting and/or nutritional oedema admitted to Moyo NRU.

Year	N	Age (months)	Sex, female	Breastfeeding (<12 months)
**2011**	**26**	22 (12, 30)	17/25 (68%)	2/2 (100%)
**2012**	**268**	23 (15, 31)	133/256 (52%)	18/28 (64%)
**2013**	**163**	23 (15, 36)	89/159 (56%)	12/19 (63%)
**2014**	**332**	24 (15, 38)	172/328 (52%)	16/25 (64%)
**2015**	**225**	24 (15, 32)	107/223 (48%)	3/6 (50%)
**2016**	**125**	23 (13, 37)	68 (54%)	5/12 (42%)
**2017**	**72**	24 (13, 38)	33 (46%)	4/4 (100%)
**2018**	**95**	20 (11, 31)	57 (60%)	14/16 (88%)
**2019**	**53**	21 (13, 29)	29/52 (56%)	7/7 (100%)
**2020**	**89**	19 (12, 32)	43 (48%)	15/18 (83%)
**2021**	**49**	23 (13, 30)	27 (55%)	10/11 (91%)
**Non-linear trend**	Intercept	-	-	74% (65, 81)
	E.D.F.	-	-	1.7
	p-value	-	-	0.020
**Linear trend**	Intercept	23 (23, 24)	53% (50-55)	70% (62, 78)
	p-value	0.072	0.81	0.0082

Median and interquartile range or n (%) presented as appropriate. Prevalence of breastfeeding was calculated in children younger than 12 months of age. Linear and non-linear trends were tested with general additive models.
