# Supplementary material for: Ten-year trends in clinical characteristics and outcome of children hospitalized with severe wasting or nutritional edema in Malawi (2011–2021): Declining admissions but worsened clinical profiles
Source: PLoS One. 2024 Dec 26;19(12):e0311534. doi: 10.1371/journal.pone.0311534 (PMC11670969; doi:10.1371/journal.pone.0311534)
Supplement: S2 Table — n (%) present severity of edema (+), (++), (+++) as defined by the World Health Organisation. Linear and non-linear trends were tested with general additive models. (PDF) [file pone.0311534.s007.pdf]

S2 Table. Trends in nutritional oedema and severity of oedema over the 10-year period.

Year	N	Oedema	Oedema levels		
			+	++	+++
**2011**	**26**	19 (73)	5 (22%)	9 (39%)	5 (22%)
**2012**	**268**	194 (72%)	43 (16%)	97 (37%)	54 (21%)
**2013**	**163**	102 (63%)	28 (18%)	53 (33%)	21 (13%)
**2014**	**332**	211 (64%)	42 (13%)	98 (31%)	71 (22%)
**2015**	**225**	126 (56%)	31 (14%)	55 (25%)	40 (19%)
**2016**	**125**	59 (47%)	12 (9.8%)	26 (21%)	21 (17%)
**2017**	**72**	27 (38%)	3 (4.5%)	16 (24%)	8 (12%)
**2018**	**95**	40 (42%)	6 (6.6%)	20 (22%)	14 (15%)
**2019**	**53**	20 (38%)	4 (9.1%)	7 (16%)	9 (20%)
**2020**	**89**	36 (40%)	9 (11%)	14 (17%)	13 (16%)
**2021**	**49**	20 (41%)	5 (10%)	9 (19%)	6 (12%)
**Non-linear trend**	Intercept	60% (57, 63)	13% (11, 15)	-	-
	E.D.F.	1.8	1.2	-	-
	p-value	<0.001	0.0028	-	-
**Linear trend**	Intercept	60% (57, 62)	13% (11, 15)	28% (25, 30)	18% (16, 20)
	p-value	<0.001	0.0013	<0.001	0.11

n (%) present severity of oedema (+), (++), (+++) as defined by the World Health Organisation.

Linear and non-linear trends were tested with general additive models.
