# Supplementary material for: Ten-year trends in clinical characteristics and outcome of children hospitalized with severe wasting or nutritional edema in Malawi (2011–2021): Declining admissions but worsened clinical profiles
Source: PLoS One. 2024 Dec 26;19(12):e0311534. doi: 10.1371/journal.pone.0311534 (PMC11670969; doi:10.1371/journal.pone.0311534)
Supplement: S3 Table — n (%) presented. Linear and non-linear trends were tested with general additive models. (PDF) [file pone.0311534.s008.pdf]

S3 Table. Trend in selected Integrated Management of Childhood Illness (IMCI) danger signs for prevalence of diarrhea, difficult breathing, dehydration, and pallor over the 10-year period in children with severe wasting and/or nutritional oedema admitted to Moyo NRU.

Year	N	Diarrhea	Difficult breathing	Dehydration	Pallor
**2011**	**26**	6/16 (38%)	1/16 (6.2%)	3/16 (19%)	0/16 (0%)
**2012**	**268**	127/261 (49%)	30/261 (11%)	39/256 (15%)	17/262 (6.5%)
**2013**	**163**	82/154 (53%)	23/152 (15%)	18/127 (14%)	13/145 (9.0%)
**2014**	**332**	111/269 (41%)	48/261 (18%)	55/258 (21%)	36/258 (14%)
**2015**	**225**	104/199 (52%)	40/196 (20%)	45/186 (24%)	25/191 (13%)
**2016**	**125**	56/121 (46%)	26/120 (22%)	20/120 (17%)	13/120 (11%)
**2017**	**72**	22/51 (43%)	10/49 (20%)	9/47 (19%)	8/50 (16%)
**2018**	**95**	38/75 (51%)	19/74 (26%)	16/72 (22%)	4/72 (5.6%)
**2019**	**53**	18/43 (42%)	11/43 (26%)	9/42 (21%)	6/42 (14%)
**2020**	**89**	27/68 (40%)	18/69 (26%)	16/65 (25%)	7/66 (11%)
**2021**	**49**	18/28 (64%)	6/27 (22%)	11/26 (42%)	7/26 (27%)
**Non-linear trend**	Intercept	-	18% (16, 20)	-	11% (9.1, 13)
	E.D.F.	-	1.7	-	1.5
	p-value	-	<0.001	-	0.053
**Linear trend**	Intercept	47% (45, 50)	18% (16, 20)	20% (18, 22)	11% (9.3, 13)
	p-value	0.81	<0.001	0.0032	0.022

n (%) presented. Linear and non-linear trends were tested with general additive models.
