# Supplementary material for: Ten-year trends in clinical characteristics and outcome of children hospitalized with severe wasting or nutritional edema in Malawi (2011–2021): Declining admissions but worsened clinical profiles
Source: PLoS One. 2024 Dec 26;19(12):e0311534. doi: 10.1371/journal.pone.0311534 (PMC11670969; doi:10.1371/journal.pone.0311534)
Supplement: S4 Table — n (%) presented. Linear and non-linear trends were tested with general additive models. (PDF) [file pone.0311534.s009.pdf]

S4 Table. Trend in selected Integrated Management of Childhood Illness (IMCI) danger signs for prevalence of cough, vomitting, and neurological signs over the 10-year period in children with severe wasting and/or nutritional oedema admitted to Moyo NRU.

Year	N	Cough	Vomiting	Neurological signs
**2011**	**26**	10/16 (62%)	4/16 (25%)	0/16 (0%)
**2012**	**268**	127/261 (49%)	102/261 (39%)	6/260 (2.3%)
**2013**	**163**	73/153 (48%)	64/154 (42%)	3/125 (2.4%)
**2014**	**332**	125/264 (47%)	99/266 (37%)	15/240 (6.2%)
**2015**	**225**	97/198 (49%)	76/199 (38%)	11/181 (6.1%)
**2016**	**125**	65/121 (54%)	45/121 (37%)	15/116 (13%)
**2017**	**72**	18/50 (36%)	15/49 (31%)	3/46 (6.5%)
**2018**	**95**	37/74 (50%)	33/75 (44%)	4/68 (5.9%)
**2019**	**53**	25/44 (57%)	19/43 (44%)	3/38 (7.9%)
**2020**	**89**	31/69 (45%)	26/68 (38%)	2/62 (3.2%)
**2021**	**49**	14/27 (52%)	14/28 (50%)	1/24 (4.2%)
**Non-linear trend**	Intercept	-	-	4.7% (3.6, 6.2)
	E.D.F.	-	-	1.9
	p-value	-	-	0.0012
**Linear trend**	Intercept	49% (46, 51)	39% (36, 42)	5.3% (4.2, 6.8)
	p-value	0.99	0.48	0.043

n (%) presented. Linear and non-linear trends were tested with general additive models.
