# Supplementary material for: Ten-year trends in clinical characteristics and outcome of children hospitalized with severe wasting or nutritional edema in Malawi (2011–2021): Declining admissions but worsened clinical profiles
Source: PLoS One. 2024 Dec 26;19(12):e0311534. doi: 10.1371/journal.pone.0311534 (PMC11670969; doi:10.1371/journal.pone.0311534)
Supplement: S5 Table — n (%) present frequency of HIV. Linear and non-linear trends were tested with general additive models. (PDF) [file pone.0311534.s010.pdf]

S5 Table. Trend in HIV reactivity and testing over the 10-year period in children with severe wasting and/or nutritional oedema admitted to Moyo NRU.

**Year**	**N**	HIV testing	HIV positive	HIV exposed
**2011**	**26**	8 (31%)	1 (3.8%)	1 (3.8%)
**2012**	**268**	95 (35%)	22 (8.2%)	10 (3.7%)
**2013**	**163**	68 (42%)	21 (13%)	1 (0.61%)
**2014**	**332**	171 (52%)	48 (14%)	14 (4.2%)
**2015**	**225**	120 (53%)	37 (16%)	14 (6.2%)
**2016**	**125**	109 (87%)	13 (10%)	11 (8.8%)
**2017**	**72**	42 (58%)	4 (5.6%)	7 (9.7%)
**2018**	**95**	55 (58%)	12 (13%)	9 (9.5%)
**2019**	**53**	38 (72%)	7 (13%)	4 (7.5%)
**2020**	**89**	55 (62%)	5 (5.6%)	7 (7.9%)
**2021**	**49**	22 (45%)	2 (4.1%)	1 (2.0%)
**Non-linear trend**	Intercept	52% (50, 55)	11% (9.6, 13)	4.9% (3.9, 6.2)
	E.D.F.	2.0	1.9	1.8
	p-value	<0.001	0.0048	0.0051
**Linear trend**	Intercept	52% (50, 55)	11% (9.9, 13)	5.1 % (4.1, 6.3)
	p-value	<0.001	0.33	0.0056

n (%) present frequency of HIV. Linear and non-linear trends were tested with general additive models.
