# Supplementary material for: Ten-year trends in clinical characteristics and outcome of children hospitalized with severe wasting or nutritional edema in Malawi (2011–2021): Declining admissions but worsened clinical profiles
Source: PLoS One. 2024 Dec 26;19(12):e0311534. doi: 10.1371/journal.pone.0311534 (PMC11670969; doi:10.1371/journal.pone.0311534)
Supplement: S6 Table — Frequencies presented as n(%). Linear and non-linear trends were tested with general additive models. (PDF) [file pone.0311534.s011.pdf]

S6 Table. Trend in vaccination reporting over the 10-year period in children with severe wasting and/or nutritional oedema admitted to Moyo NRU.

		Vaccination		
**Year**	**N**	Complete n (%)	Missed n (%)	Unrecorded n (%)
**2011**	**26**	14 (54%)	0 (0%)	12 (46%)
**2012**	**268**	216 (81%)	10 (3.7%)	42 (16%)
**2013**	**163**	114 (70%)	7 (4.3%)	42 (26%)
**2014**	**332**	170 (51%)	47 (14%)	115 (35%)
**2015**	**225**	134 (60%)	23 (10%)	68 (30%)
**2016**	**125**	75 (60%)	13 (10%)	37 (30%)
**2017**	**72**	29 (40%)	9 (13%)	34 (47%)
**2018**	**95**	37 (39%)	25 (26%)	33 (35%)
**2019**	**53**	14 (26%)	16 (30%)	23 (43%)
**2020**	**89**	32 (36%)	20 (22%)	37 (42%)
**2021**	**49**	17 (35%)	8 (16%)	24 (49%)
**Non-linear trend**	Intercept	57% (55, 60)	11% (83, 88)	31% (28, 33)
	E.D.F.	1.7	1.9	1.4
	p-value	<0.001	<0.001	<0.001
**Linear trend**	Intercept	57% (55, 60)	11% (9.2, 13)	31% (28, 33)
	p-value	<0.001	<0.001	<0.001

Frequencies presented as n(%). Linear and non-linear trends were tested with general additive models.
