# Supplementary material for: Ten-year trends in clinical characteristics and outcome of children hospitalized with severe wasting or nutritional edema in Malawi (2011–2021): Declining admissions but worsened clinical profiles
Source: PLoS One. 2024 Dec 26;19(12):e0311534. doi: 10.1371/journal.pone.0311534 (PMC11670969; doi:10.1371/journal.pone.0311534)
Supplement: S7 Table — Frequencies presented as n(%). Linear and non-linear trends were tested with general additive models. Antibiotic classification as per WHO-Aware framework relating to potential of contributing to antibiotic resistance. WHO AWaRe classification groups antibiotics into ‘Access’ (broad spectrum antibiotics with a lower risk of resistance, e.g., benzathine penicillin, amoxicillin and chloramphenicol) and ‘Watch’ (broad spectrum with higher risk of selection of bacterial resistance, e.g., ceftriaxone, ciprofloxacin and meropenem). (PDF) [file pone.0311534.s012.pdf]

S7 Table. Trends in antibiotic usage over the 10-year period in children with severe wasting and/or nutritional oedema admitted to Moyo NRU.

Year	N	Access	Watch
**2011**	**26**	26 (100%)	6 (23%)
**2012**	**268**	264 (99%)	49 (18%)
**2013**	**163**	154 (94%)	43 (26%)
**2014**	**332**	308 (93%)	101 (30%)
**2015**	**225**	219 (97%)	63 (28%)
**2016**	**125**	120 (96%)	31 (25%)
**2017**	**72**	62 (86%)	21 (29%)
**2018**	**95**	87 (92%)	23 (24%)
**2019**	**53**	52 (98%)	10 (19%)
**2020**	**89**	75 (84%)	34 (38%)
**2021**	**49**	40 (82%)	11 (22%)
**Non-linear trend**	Intercept	-	26% (24, 28)
	E.D.F.	-	1.6
	p-value	-	0.1078
**Linear trend**	Intercept	95% (93, 96)	26% (24, 28)
	p-value	<0.001	0.083

Frequencies presented as n(%). Linear and non-linear trends were tested with general additive models. Antibiotic classification as per WHO-Aware framework relating to potential of contributing to antibiotic resistance. WHO AWaRe classification groups antibiotics into 'Access' (broad spectrum antibiotics with a lower risk of resistance) and 'Watch' (broad spectrum with higher risk of selection of bacterial resistance).
