# Supplementary material for: Ten-year trends in clinical characteristics and outcome of children hospitalized with severe wasting or nutritional edema in Malawi (2011–2021): Declining admissions but worsened clinical profiles
Source: PLoS One. 2024 Dec 26;19(12):e0311534. doi: 10.1371/journal.pone.0311534 (PMC11670969; doi:10.1371/journal.pone.0311534)
Supplement: S8 Table — Median and interquartile range or n (%) presented as appropriate. Linear and non-linear trends were tested with general additive models. (PDF) [file pone.0311534.s013.pdf]

S8 Table. Trends in mortality, readmission and time to death or discharge over the 10-year period in children with severe wasting and/or nutritional oedema admitted to Moyo NRU.

Year	N	Deaths	Days-to-death	Days-to-discharge	Readmission
2011	26	8 (31%)	4.5 (2.0, 14)	7.0 (6.0, 10)	1 (3.8%)
2012	268	70 (26%)	3.0 (1.0, 6.0)	7.0 (6.0, 10)	10 (3.7%)
2013	163	39 (24%)	4.0 (1.0, 7.5)	7.0 (6.0, 10)	13 (8.0%)
2014	332	68 (20%)	5.0 (2.0, 7.0)	7.0 (5.0, 10)	34 (10%)
2015	225	59 (26%)	4.0 (2.0, 10)	6.5 (5.0, 8.0)	19 (8.4%)
2016	125	27 (22%)	3.0 (2.0, 4.0)	7.0 (5.0, 10)	8 (6.4%)
2017	72	14 (19%)	3.0 (2.2, 6.2)	5.0 (4.0, 7.0)	17 (24%)
2018	95	16 (17%)	2.5 (2.0, 4.2)	6.0 (3.0, 8.0)	24 (25%)
2019	53	14 (26%)	2.0 (1.0, 3.0)	6.5 (5.2, 9.0)	13 (25%)
2020	89	22 (25%)	2.0 (1.0, 4.8)	5.0 (4.2, 8.0)	10 (11%)
2021	49	9 (18%)	2.0 (1.0, 3.0)	2	11 (22%)
**Non-linear trend**	Intercept (95%CI)	-	-	-	9.6% (8.2, 11)
	E.D.F.	-	-	-	1.8
	p-value	-	-	-	<0.001
**Linear trend**	Intercept (95%CI)	23% (21, 25)	4.6 (4.2, 5.1)	7.5 (7.3, 7.8)	9.8% (8.4, 12)
	p-value	0.23	0.0093	<0.001	<0.001

Median and interquartile range or n (%) presented as appropriate. Linear and non-linear trends were tested with general additive models.
