# Supplementary material for: Ten-year trends in clinical characteristics and outcome of children hospitalized with severe wasting or nutritional edema in Malawi (2011–2021): Declining admissions but worsened clinical profiles
Source: PLoS One. 2024 Dec 26;19(12):e0311534. doi: 10.1371/journal.pone.0311534 (PMC11670969; doi:10.1371/journal.pone.0311534)
Supplement: S9 Table — Results from logistic regression analysis presenting odds ratios (OR) and 95% confidence intervals (95%CI). WHZ, weight-for-height/length z-score. (PDF) [file pone.0311534.s014.pdf]

S9 Table. Clinical features associated with readmission of children with severe wasting and/or nutritional oedema admitted to MOYO Nutritional Rehabilitation Unit across the 10-year period.

Clinical feature	Readmission % [95%CI]	OR [95%CI] adjusted[1]	*p*	OR [95%CI] adjusted[2]	*p*
**WHZ**					
≥ -3.5	11 [8, 14]	*Ref*		-	
< -3.5	14 [11, 18]	1.49 [0.97, 2.29]	n.s.	-	
**Age**					
≥ 5y	15 [10, 21]	*Ref*		*Ref*	
≥ 2 & < 5 years	11 [9, 15]	0.80 [0.46, 1.40]	n.s.	1.00 [0.50, 2.10]	n.s.
< 2 years	9 [7, 12]	0.62 [0.36, 1.10]	n.s.	0.55 [0.28, 1.13]	n.s.
**Oedema**					
No	15 [12, 19]	*Ref*		*Ref*	
Yes	7 [6, 10]	0.44 [0.30, 0.65]	***	0.37 [0.20, 0.66]	**
**Dehydration**					
No	10 [9, 13]	*Ref*		*Ref*	
Yes	9 [6, 15]	0.85 [0.45, 1.50]	n.s.	1.13 [0.56, 2.14]	n.s.
**Diarrhoea**					
No	11 [9, 14]	*Ref*		*Ref*	
Yes	8 [6, 11]	0.71 [0.46, 1.10]	n.s.	0.89 [0.51, 1.53]	n.s.
**Vomit**					
No	10 [8, 12]	*Ref*		*Ref*	
Yes	10 [7, 14]	1.01 [0.65, 1.57]	n.s.	1.32 [0.76, 2.27]	n.s.
**Cough**					
No	8 [6, 11]	*Ref*		*Ref*	
Yes	12 [9, 15]	1.42 [0.93, 2.20]	n.s.	1.0.5 [0.61, 1.82]	n.s.
**Difficult breathing**					
No	9 [7, 11]	*Ref*		*Ref*	
Yes	14 [9, 20]	1.50 [0.88, 2.48]	n.s.	1.40 [0.71, 2.64]	n.s.
**HIV**					
No	9.7 [8.1, 12]	*Ref*		*Ref*	
Yes	17 [11, 24]	1.79 [1.05, 2.96]	0.026	1.59 [0.79, 3.01]	n.s.

Results from logistic regression analysis presenting odds ratios (OR) and 95% confidence intervals (95%CI). WHZ, weight-for-height/length z-score.
