# Supplementary material for: Ten-year trends in clinical characteristics and outcome of children hospitalized with severe wasting or nutritional edema in Malawi (2011–2021): Declining admissions but worsened clinical profiles
Source: PLoS One. 2024 Dec 26;19(12):e0311534. doi: 10.1371/journal.pone.0311534 (PMC11670969; doi:10.1371/journal.pone.0311534)
Supplement: S10 Table — Results from competitive risk analysis presenting unadjusted or age- and WHZ- adjusted daily risk of mortality as odds ratios (OR) and 95% confidence intervals (95%CI). WHZ, weight-for-height/length z-score. (PDF) [file pone.0311534.s015.pdf]

S10 Table. Clinical features associated with daily probability of mortality in children with severe wasting and/or nutritional oedema admitted to MOYO Nutritional Rehabilitation Unit across the 10-year period.

		**Mortality**			
		Unadjusted Est. 95%CI	*p*	Adjusted Est. 95%CI	*p*
**Age and WHZ**	Age, months			1.00 [0.982, 1.02]	0.86
	WHZ			0.626 [0.536, 0.73]	<0.0001
	Age x WHZ			1.01 [1.00, 1.01]	0.035
	Observations			821	
**Dehydration**	Dehydration	2.17 [1.69, 2.78]	<0.0001	1.44 [0.997, 2.09]	0.052
	Age, months	-	-	0.983 [0.974, 0.992]	<0.001
	WHZ	-	-	0.717 [0.645, 0.797]	<0.0001
	Observations	1196		646	
**Diarrhoea**	Diarrhoea	1.38 [1.1, 1.72]	0.0048	1.03 [0.744, 1.42]	0.87
	Age, months	-	-	0.982 [0.973, 0.991]	<0.001
	WHZ	-	-	0.724 [0.658, 0.798]	<0.0001
	Observations	1265		681	
**Dehydration and diarrhoea**	Both	2.31 [1.78, 2.99]	<0.0001	1.46 [0.981, 2.17]	0.062
	Age, months	-	-	0.983 [0.974, 0.993]	<0.001
	WHZ	-	-	0.716 [0.644, 0.796]	<0.0001
	Observations	1181		634	
**Oedema**	Oedema	0.661 [0.532, 0.822]	<0.001	1.40 [1.01, 1.95]	0.047
	Age, months	-	-	0.982 [0.974, 0.991]	<0.001
	WHZ	-	-	0.679 [0.615, 0.751]	<0.0001
	Observations	1407		785	
**Vomitting**	Vomitting	1.41 [1.13, 1.77]	0.0027	1.43 [1.04, 1.97]	0.028
	Age, months	-	-	0.982 [0.974, 0.991]	<0.001
	WHZ	-	-	0.731 [0.644, 0.806]	<0.0001
	Observations	1260		679	
**Difficulty breathing**	Difficulty breathing	1.71 [1.32, 2.22]	<0.0001	1.12 [0.75, 1.65]	0.59
	Age, months	-	-	0.983 [0.974, 0.991]	<0.001
	WHZ	-	-	0.729 [0.661, 0.805]	<0.0001
	Observations	1248		674	
**Cough**	Cough	1.35 [1.08, 1.69]	0.0090	1.08 [0.786, 1.49]	0.63
	Age, months	-	-	0.982 [0.973, 0.991]	<0.001
	WHZ	-	-	0.720 [0.652, 0.794]	<0.0001
	Observations	1257		678	

Results from competitive risk analysis presenting unadjusted or age- and WHZ- adjusted daily risk of mortality as odds ratios (OR) and 95% confidence intervals (95%CI). WHZ, weight-for-height/length z-score.
