# Supplementary material for: Ten-year trends in clinical characteristics and outcome of children hospitalized with severe wasting or nutritional edema in Malawi (2011–2021): Declining admissions but worsened clinical profiles
Source: PLoS One. 2024 Dec 26;19(12):e0311534. doi: 10.1371/journal.pone.0311534 (PMC11670969; doi:10.1371/journal.pone.0311534)
Supplement: S11 Table — Results from competitive risk analysis presenting unadjusted or age- and WHZ- adjusted daily risk of discharge as odds ratios (OR) and 95% confidence intervals (95%CI). WHZ, weight-for-height/length z-score. (PDF) [file pone.0311534.s016.pdf]

S11 Table. Clinical features associated with daily probability of discharge in children with severe wasting and/or nutritional oedema admitted to MOYO Nutritional Rehabilitation Unit across the 10-year period.

		Discharge			
		Unadjusted		Adjusted	
		Est. 95%CI	*p*	Est. 95%CI	*p*
**Age and WHZ**	Age, months			0.998 [0.994, 1.003]	0.46
	WHZ			1.226 [1.160, 1.295]	<0.0001
	Age x WHZ			0.998 [0.997, 0.999]	0.00049
	Observations			821	
**Dehydration**	Dehydration	0.65 [0.546, 0.775]	<0.0001	0.89 [0.712, 1.11]	0.31
	Age, months	-	-	1.00 [1.00, 1.01]	0.0012
	WHZ	-	-	1.14 [1.094, 1.19]	<0.0001
	Observations	1196		646	
**Diarrhoea**	Diarrhoea	0.843 [0.748, 0.951]	0.0055	0.952 [0.811, 1.12]	0.55
	Age, months	-	-	1.005 [1.003, 1.01]	<0.0001
	WHZ	-	-	1.15 [1.101, 1.19]	<0.0001
	Observations	1265		681	
**Dehydration and diarrhoea**	Both	0.609 [0.502, 0.739]	<0.0001	0.862 [0.670, 1.11]	0.25
	Age, months	-	-	1.004 [1.00, 1.01]	<0.001
	WHZ	-	-	1.142 [1.10, 1.19]	<0.0001
	Observations	1181		634	
**Dehydration and diarrhoea**	Both	0.666 [0.435, 1.02]	0.061	0.793 [0.467, 1.35]	0.39
	Dehydration only	0.912 [0.630, 1.32]	0.63	1.084 [0.689, 1.70]	0.71
	Diarrhoea only	0.997 [0.872, 1.14]	0.97	1.011 [0.844, 1.21]	0.91
	Age, months	-	-	1.004 [1.002, 1.01]	<0.001
	WHZ	-	-	1.14 [1.096, 1.19]	<0.001
	Observations	1181		634	
**Oedema**	Oedema	1.2 [1.06, 1.35]	0.0031	0.797 [0.662, 0.961]	0.017
	Age, months	-	-	1.005 [1.002, 1.007]	<0.001
	WHZ	-	-	1.182 [1.130, 1.238]	<0.0001
	Observations	1407		785	
**Vomitting**	Vomitting	0.802 [0.708, 0.909]	<0.001	0.794 [0.673, 0.938]	0.0067
	Age, months	-	-	1.005 [1.003, 1.008]	<0.0001
	WHZ	-	-	1.14 [1.09, 1.19]	<0.0001
	Observations	1260		679	
**Difficulty breathing**	Difficulty breathing	0.762 [0.641, 0.905]	0.0020	0.991 [0.802, 1.22]	0.93
	Age, months	-	-	1.005 [1.002, 1.01]	<0.0001
	WHZ	-	-	1.14 [1.10, 1.19]	<0.0001
	Observations	1248		674	
**Cough**	Cough	0.843 [0.748, 0.95]	0.0053	0.939 [0.8, 1.10]	0.44
	Age, months	-	-	1.005 [1.00, 1.01]	<0.001
	WHZ	-	-	1.15 [1.10, 1.20]	<0.0001
	Observations	1257		678	

Results from competitive risk analysis presenting unadjusted or age- and WHZ- adjusted daily risk of discharge as odds ratios (OR) and 95% confidence intervals (95%CI). WHZ, weight-for-height/length z-score.
