# Supplementary material for: Ten-year trends in clinical characteristics and outcome of children hospitalized with severe wasting or nutritional edema in Malawi (2011–2021): Declining admissions but worsened clinical profiles
Source: PLoS One. 2024 Dec 26;19(12):e0311534. doi: 10.1371/journal.pone.0311534 (PMC11670969; doi:10.1371/journal.pone.0311534)
Supplement: Attachment — Submitted filename: Re.PLOS_TRENDS_docx.docx [file pone.0311534.s018.docx]

Re: Ten-year trends in clinical characteristics and outcome of children hospitalized with

severe wasting or nutritional oedema in Malawi (2011-2021): Declining admissions but

worsened clinical profiles

Overall comments

There should be a flow chart illustrating patients recruited/enrolment as appropriate

Abstract “

Results-line 45 mentioned anthropometry-indicate which parameters you assessed.

Line 47 “HIV testing increased but detection remained at 11%.” Is this annually or overall? Clarify

Introduction

Methods

“Medical files were accessed from 8th of June 2020, to 20th of May, 2022” Is this for retrieval of the files?

“Files were stored by date of admission, thus, starting from a randomly selected file, every third record was marked for inclusion” please provide ref/any justification for your decision.

“Research staff” how many?

“demographics (i.e., age, sex, breastfeeding in children < 12 months)” Breastfeeding is not demographics and can it described what you meant breastfeeding less than 12? Still breasting/EBM

“‘Access’ (broad-spectrum with a lower risk of resistance) and ‘Watch’ (broad-spectrum with higher risk of selection for bacterial resistance)” Provide examples of these antibiotics from cases.

“Implausible anthropometric values of WHZ, HAZ and WAZ were filtered if exceeded +4 or fell below -10 z-scores” how many ? provide the n

Results

“From 2011 to 2021, Moyo NRU had 4494 admissions, of which 1498 (33%) were randomly selected for inclusion in this retrospective analysis” A bit difficult to understand while the number sampled was not more than this considering the high number of missing variables and incomplete data? Kindly provide rationale for this?

Table 1; Insert exact number of available anthropometry rather indicating missing

[MUAC, WHZ, WAZ, HAZ], What the access and watch group of antibiotics with examples.

“Missingness in anthropometry was undocumented in approximately 40% of health records” this is the greatest limitation of this study and is missing the study limitation, kindly provide exact number that are using.

“This decline coincided with an increase in unreported vaccination status of patients” I can not locate this in the results?

(i.e. 346 mortality cases out of 1498 children)-This appear incorrect since close to 40% of children did not have anthropometry for proper classification/inclusion/exclusion

Table 2-can you clarify the models, model 2 did not include anthropometry???

Discussion

“However, there was a significant 40% reduction in the prevalence of nutritional edema” insert n before the percent ?

“To the best of our knowledge, this is the first study to cover a 10-year period and highlight changing trends in clinical presentation” This statement should be re-casted, this data has close to half missing with no details of what is missing in the anthropometry

“We were unable to capture laboratory or radiological findings or include specific chronic conditions associated with malnutrition like tuberculosis due to low recorded prevalence” How low is this? This should have been reported, it is an important co-morbidity that may influence outcomes

Figures 1 to 4 are poor qualities and hard to read, kindly revise to enhance readability

S1 Table: The age and sex has non-linear trend, what inform the choice to report non-linear vs linear trend

S2 Table: Again non-linear trend was not reported for oedema grade 2 and 3, even if it is not significant, this should be reported for consistency and if it is not done, a footnote should be added indicating why it was not done.

S3 Table; Same comments as S2 Table: Non-linear trend not reported for some variables?

S7 Table: Same comments

S8 Table: ? Non-linear trends? Also, the number of some variables are small? 26 for a whole year?
